# Core fucosylated glycan-dependent inhibitory effect of QSOX1-S on invasion and metastasis of hepatocellular carcinoma

**DOI:** 10.1038/s41420-019-0164-8

**Published:** 2019-04-03

**Authors:** Xiao-Fei Zhang, Ji Wang, Hu-Liang Jia, Wen-Wei Zhu, Lu Lu, Qing-Hai Ye, Peter J. Nelson, Yi Qin, Dong-Mei Gao, Hai-Jun Zhou, Lun-Xiu Qin

**Affiliations:** 1 Department of General Surgery, Huashan Hospital & Cancer Metastasis Institute, Fudan University, Shanghai, China; 2 Department of General Surgery, Affiliated Hospital of Xuzhou Medical College, Xuzhou, Jiangsu Province China; 3 Liver Cancer Institute & Zhongshan Hospital, Fudan University, Shanghai, China; 4 Key Laboratory of Carcinogenesis & Cancer Invasion, Ministry of Education, Shanghai, China; 5 Medizinische Klinik und Poliklinik IV, University of Munich, Munich, Germany; 6 Pancreatic Cancer Institute, Fudan University, 200032 Shanghai, China; 7 Institute of Biomedical Sciences, Fudan University, Shanghai, China

## Abstract

The goal of the present study was to identify glycoproteins associated with the postoperative relapse of hepatocellular carcinoma (HCC) and to investigate their potential role in HCC metastasis. A method for quantitating N-glycoproteome was used to screen for, and identify, recurrence-related N-linked glycoproteins from 100 serum samples taken from patients with early-stage HCC. The prognostic significance of candidate glycoproteins was then validated in 193 HCC tissues using immunohistochemical staining. Serum core fucosylated quiescin sulfhydryl oxidase 1 (cf-QSOX1) was identified as a leading prognostic glycoprotein that significantly correlated with HCC recurrence. Patients with high serum cf-QSOX1 levels had a significantly longer time to recurrence (TTR) as compared with those with low serum cf-QSOX1. As was seen with serum cf-QSOX1, QSOX1 in HCC tissues was further shown to be significantly associated with good patient outcome. Gain-functional and loss-functional analyses of QSOX1-S were performed in vitro and in vivo. QSOX1-S overexpression significantly increased in vitro apoptosis, but decreased the invasive capacity of HCC cells, and reduced lung metastasis in nude mice models bearing human HCC. Furthermore, overexpression of a mutant version of QSOX1-S, which had eliminated the core-fucosylated glycan at Asn-130, showed no demonstrable effect on invasion or metastasis of HCC cells. Our study suggests that serum cf-QSOX1-S and tumor QSOX1 levels are helpful for predicting recurrence in HCC patients, and its core-fucosylated glycan at Asn-130 is critical for the inhibitory effects of QSOX1-S on invasion and metastasis of HCC

## Introduction

Hepatocellular carcinoma (HCC) is one of the most common and aggressive human malignancies worldwide^1,2^. The general prognosis of this disease is still extremely poor despite improved clinical diagnosis and treatment strategies that have emerged during the past few decades. The poor patient outcome seen is largely attributed to the high frequency of metastatic recurrence after curative treatments^3–5^. An expansion of the prognostic markers available, and identifying pathophysiologic mechanisms linked to metastatic recurrence, would help in the development of adjuvant therapies after resection and also provide potential targets for combating HCC metastasis.

Glycosylation occurs as a post-transcriptional modification of proteins during their biogenesis. Glycoproteins carry one or more glycans that are covalently attached to the polypeptide backbone, this occurs usually via nitrogen or oxygen linkages, referred to as N-glycans or O-glycans, respectively^6^. It has been suggested that N-glycan β1,6-GlcNAc branching structures, bisecting GlcNAc, and core fucose have been linked to cancer biology^7^. The accumulating data strongly suggests that glycosylation may play fundamental roles in key pathological steps of tumor development and progression^8–12^. To this end, glycosylation has been shown to be involved in tumor cell–cell adhesion, cell–matrix interaction, cancer metabolism, and tumor immune surveillance^6^. More recently, an aberrant glycosylation that helps drive melanoma metastasis has been reported. The study also strongly underscored the urgent need for more systematic analyses of glycosylation in clinical tumor samples^13^.

Many established tumor biomarkers including: alpha-fetoprotein (AFP), carcinomaembryonic antigen (CEA), CA125 and prostate-specific antigen (PSA), are glycosylated proteins^14–17^. Glycan biomarkers detectable in serum or plasma could potentially enhance cancer diagnosis and prognosis. For example, serum AFP is a marker used in the diagnosis of HCC, but its relatively low specificity for discrimination between HCC and the benign liver diseases has limited its broad clinical application. By contrast, serum fucosylated AFP‐L3 fraction has been shown to help distinguish liver fibrosis from HCC^6,18^.

Since the majority of blood glycoproteins are synthesized in the liver, serum, or plasma represent a good source for identifying potential glycoprotein biomarkers for characterizing liver diseases^8,19^. The goal of the present study was to identify potential serum glycoproteins linked to HCC recurrence. New systematic glycoproteomic approaches now allow the discovery of specific protein glycosylation information occurring in cancer. In a previous study, we described the development of a method for quantifying the N-glycoproteome in blood samples using lectin affinity chromatography combined with enzyme-catalyzed ^18^O_3−_ or ^16^O_3−_ labeling. The potential feasibility of using this approach for the identification of disease-related N-linked glycoproteins using serum samples from HCC patients and healthy individuals was proposed^20^.

In the present study, our previous method was used to screen for HCC recurrence-related N-linked glycoproteins in the serum of HCC patients. A major challenge in the treatment of HCC is the identification of patients who are at a greater risk for tumor recurrence after treatment, particularly for patients with early stage disease who do not show significant vascular invasion, regional lymph node, or distant metastasis^21^. We screened serum samples, and identified a potential recurrence-related N-linked glycoprotein from HCC patients with BCLC 0 or A stage disease (cohort A). Serum core-fucosylated quiescin sulfhydryl oxidase 1 (cf-QSOX1) was found to be significantly associated with postoperative recurrence of HCC, and serum QSOX1 was shown to be completely represented by the 67 kDa short isoform of QSOX1 (QSOX1-S). We further validated the prognostic significance of QSOX1 in tumor tissues for HCC patients after curative resection. A significant positive correlation was found between serum cf-QSOX1 and tumor expressed QSOX1. The functional roles of QSOX1-S and its glycosylation in HCC metastasis were further investigated. We show that QSOX1-S expression can inhibit HCC tumor invasion and metastasis. Glycosylation at residue Asn-130 was further shown to be required for the inhibitory effects on invasion and metastasis of HCC. A flowchart of experimental plan is shown in Supplemental Fig. S1.

## Results

### High serum levels of core-fucosylated QSOX1-S correlates with long time to recurrence (TTR) of patients after HCC resection

N-glycan structures on glycoproteins can be specifically recognized and bound by a given lectin^22^. A lectin microarray analysis was performed on 40 tumor tissue samples from patients who had undergone curative surgical resections for HCC (set A, *n* = 40) to identify potential glycan structures linked to metastatic recurrence of HCC. Glycoproteins from the tumor tissues that could bind lectin ConA, LCA, WGA, or MPL, were found to be significantly differentially expressed between patients who had undergone postoperative HCC recurrence (recurrence group) and those without tumor-recurrence (non-recurrence group) during the follow-up period (Supplemental Fig. S2A and B).

**Fig. 1:**
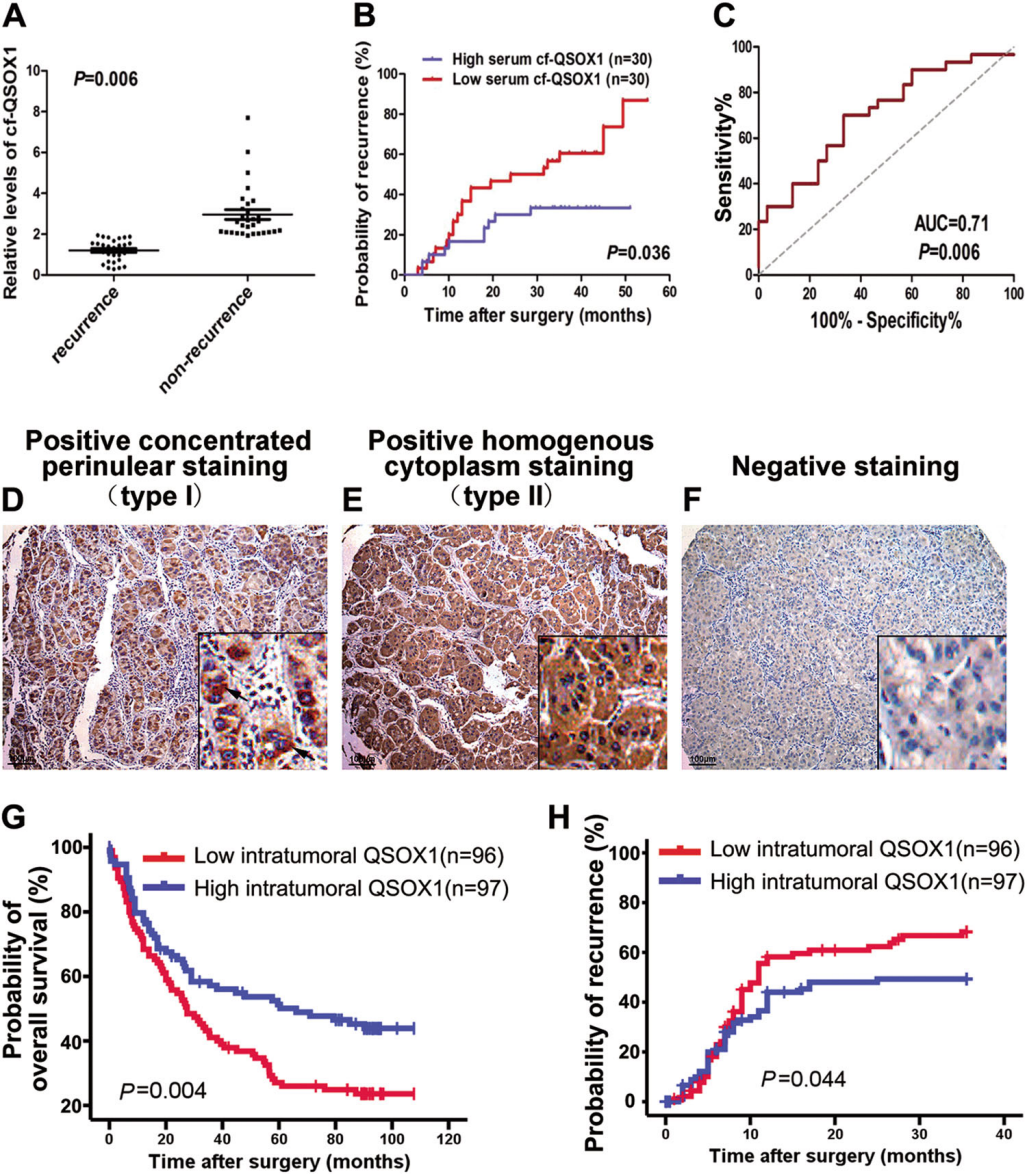
Both high serum cf-QSOX1 and tumorous QSOX1 levels correlate with better prognosis of patients with HCC. **a** The serum glycoproteins from patients of set C were enriched by LCA lectin affinity chromatography followed by Western blot against QSOX1. Serum cf-QSOX1 levels in HCC recurrence (*n* = 28) and HCC non-recurrence group (*n* = 32) were compared. **b** Kaplan–Meier curves for time to recurrence (TTR) in HCC patients of set C according to serum cf-QSOX1 level. The median expression level of serum cf-QSOX1 was used as the cutoff value. **c** Receiver operating characteristic (ROC) analysis for serum cf-QSOX1. **d–f** Sections for representative type I staining and type II staining and negative staining of QSOX1 in tumor tissues are shown (bar, 100 μm). Kaplan–Meier curves show the overall survival (**g**) and time to recurrence (**h**) in HCC patients from cohort B according to tumorous QSOX1 densities. The median QSOX1 density was used as the cutoff for the definition of subgroups. Values are expressed as the mean ± SD

Based on these results, we then sought to identify specific recurrence-related N-linked glycoproteins from the subgroups of ConA-combinable, LCA-combinable or WGA-combinable glycoproteins by using serum samples from a second cohort of 40 patients (set B, including 20 patients with HCC recurrence and 20 patients without HCC recurrence) based on the strategy shown in Supplemental Fig. S3, and previously described by our group^20^. The method is comprised of four steps: digestion of glycoproteins into glycopeptides; lectin affinity chromatography; enzyme-catalyzed ^18^O_3_− or ^16^O_3_− labeling; and mass spectrum analyses. A total of 57 ConA-combinable, LCA-combinable, or WGA-combinable glycoproteins were identified to be differentially expressed between the HCC recurrence and non-recurrence groups (Supplemental Table S1). Among these glycoproteins, LCA-combinable QSOX1, ConA-combinable Ceru, and LCA-combinable LG3BP were selected to further confirm their association with HCC recurrence. Their N-glycosylation sites and mass spectrum information are provided in Supplemental Table S2. To further validate the three recurrence-related glycoproteins, specific lectin affinity chromatography of glycoproteins was performed followed by Western blotting using serum samples from a third cohort of 60 HCC patients (set C). Because LCA lectin can specifically bind core (α1–6)-fucosylated glycoproteins, core-fucosylated QSOX1 (cf-QSOX1) was used to refer to LCA-combinable QSOX1 throughout the rest of this article. QSOX1 has two distinct isoforms, a long QSOX1 (QSOX1-L, 747 aa) and a short QSOX1 (QSOX1-S, 604 aa)^23,24^. As shown in Supplemental Fig. S4, serum cf-QSOX1 was only found in the 67 kDa position, suggesting that the serum cf-QSOX1 is comprised of QSOX1-S. The relative expression levels of serum cf-QSOX1 from the HCC recurrence group were significantly lower to those seen in the HCC non-recurrence group (Fig. 1a). Kaplan–Meier analyses using the log-rank test revealed that serum cf-QSOX1 levels were significantly associated with TTR (*P* = 0.036) when the median expression level of serum cf-QSOX1 was used as the cutoff value (Fig. 1b). Patients with low serum cf-QSOX1 levels had a significantly shorter TTR as compared to those showing high cf-QSOX1. High serum cf-QSOX1 levels appeared to be associated with a longer overall survival, although it did not reach statistical difference (Supplemental Fig. S5). Levels of the two other glycoproteins tested were not found to be significantly associated with TTR or OS in these patients (Supplemental Table S3).

Univariate and multivariate analyses of the prognostic abilities of serum cf-QSOX1 were performed. In univariate analysis, serum cf-QSOX1 and liver cirrhosis were significantly associated with TTR. In multivariate analyses, the prognostic values of cf-QSOX1 for TTR were independent from all other clinical variables tested (Table 1). The predictive value of serum cf-QSOX1 was then studied by receiver operating characteristic (ROC) analysis. The area under the curve of serum cf-QSOX1 was 0.71 (95% confidence interval, 0.57–0.84; *P* = 0.006) for recurrence (Fig. 1c). These results indicate that serum cf-QSOX1 negatively correlates with recurrence of HCC in patients with early stage disease, and is therefore a potential biomarker for predicting HCC recurrence.

**Table 1:** Univariate and multivariate Cox regression analyses of the serum core-fucosylated QSOX1 for time to recurrence (TTR) of patients in the set C (*N* = 60) of cohort A

Variables in the equation	Stratification standard	TTR			
		*p-*Value	Hazard ratio	95% CI	
				Lower	Upper
*Univariate analysis*					
Sex	Male vs. female	0.591	1.5	0.4	6.3
Age	50 vs. ≤50	0.317	0.7	0.3	1.4
AFP	20 vs. ≤20 ng/ml	0.896	1.1	0.5	2.2
Liver cirrhosis	Microndular vs. macrondular	**0.042**	2.3	1.0	4.9
Tumor size	<2 vs. ≥2 cm	0.902	1.1	0.5	2.4
Microvascular invasion	Yes vs. no	0.536	1.5	0.4	5.1
Tumor encapsulation	Yes vs. no	0.524	1.3	0.6	2.6
Tumor differentiation	I–II vs. III–IV	0.761	1.1	0.5	2.4
BCLC stage	0 vs. A	0.902	1.1	0.5	2.4
Okuda stage	I vs. II	0.179	4.0	0.5	30.8
CLIP score	0 + 1 vs. 2 + 3	0.233	3.4	0.5	25.9
cf-QSOX1	High vs. Low	**0.042**	0.5	0.2	1.0
*Multivariate analysis*					
cf-QSOX1	High vs. low	**0.047**	0.5	0.2	1.0
Liver cirrhosis	Microndular vs. macrondular	**0.047**	2.2	1.0	4.8

*95% CI* 95% confidence interval, *AFP* α-fetoprotein, *cf-QSOX1* core-fucosylated QSOX1.

The meaning of the bold *P* values is that they are under 0.05 and statistically significant

The proportion of cf-QSOX1 in total serum QSOX1 was analyzed in eight patients from cohort C. Serum cf-QSOX1 was found to account for a substantial percentage of total serum QSOX1 (90.75 ± 4.62%) (Supplemental Fig. S6).

### QSOX1 levels in tumor tissues are associated with HCC patients prognosis

Since a specific antibody directed against cf-QSOX1 was unavailable, the expression and distribution of total QSOX1 in HCC tissues was investigated by immunohistochemistry (IHC). Tumor tissue samples from 193 HCC patients (cohort B) were used for this analysis. As shown in Fig. 1d–f, QSOX1 staining was strongly positive for the tumor cells. Two QSOX1 immunostaining patterns, a concentrated perinuclear staining (type I) and a homogenous cytoplasm staining (type II) were found in the tumor cells (Fig. 1d, e). Tumor QSOX1 expression did not correlate with any clinicopathologic feature (Supplemental Table S4). Using the median QSOX1 densities as the cutoff for the definition of subgroups, Kaplan–Meier analyses showed that high tumor QSOX1 densities were significantly associated with a longer OS (Fig. 1g), and TTR within the first-3 years after operation (Fig. 1h). However, tumor QSOX1 densities did not correlate with TTR for overall follow-up time (Supplemental Fig. S7). The 1-year, 2-year, and 3-year recurrence probabilities of the low- QSOX1 group were 58.7%, 62%, and 68.7%, respectively, were significantly increased as compared to the high-QSOX1 group (43.3%, 47.3%, and 48.7%, respectively; *P* = 0.044) (Fig. 1h). These results suggest that tumor QSOX1 correlates with early recurrence of HCC, likely resulting from metastatic spread.

Using univariate analysis, tumor QSOX1, HBsAg, microvascular invasion, tumor differentiation, and tumor encapsulation were found to be significantly associated with OS during follow-up studies; while tumor QSOX1, HBsAg, and liver cirrhosis correlated with TTR during the 3-year follow up after surgery. In multivariate analyses, the prognostic values of tumor QSOX1 for OS and 3-year-TTR follow up were independent of the other clinical variables tested (Table 2).

**Table 2:** Univariate and multivariate Cox regression analyses of the tumorous QSOX1 for time to recurrence (TTR) or overall survival (OS) of patients in the cohort B (*N* = 193)

Variables	TTR (first 3-year-follow up)		OS (full follow-up)	
	Hazard ratio (95% CI)^a^	*p*-Value	Hazard ratio (95% CI)	*p*-Value
*Univariate analysis*				
Tumorous QSOX1 (high vs. low)	0.6(0.4–0.9)	**0.024**	0.6(0.4–0.9)	**0.007**
Sex (male vs. female)	0.9(0.5–1.4)	0.590	1.0(0.6–1.6)	0.928
Age (>51 vs. ≤51 years)	1.1(0.7–1.6)	0.756	1.1(0.8–1.6)	0.547
HBsAg (positive vs. negative)	7.2(1.8–29.4)	**0.006**	2.3(1.1–5.0)	**0.029**
AFP (>200 vs. ≤200 ng/ml)	1.3(0.9–2.1)	0.187	1.3(0.9–1.9)	0.208
ALT (>75 vs. ≤75 U/l)	1.5(0.8–2.7)	0.244	1.4(0.8–2.5)	0.187
Tumor size (>3 vs. ≤3 cm)	1.3(0.9–2.0)	0.164	1.4(1.0–2.0)	0.089
Tumor number (multi vs. single)	0.6(0.2–1.6)	0.310	1.6(0.8–3.2)	0.220
Tumor encapsulation (no vs. yes)	1.3(0.9–2.0)	0.153	1.5(1.0–2.1)	**0.035**
Tumor differentiation (I–II vs. III–IV)	1.4(0.9–2.1)	0.170	1.7(1.1–2.4)	**0.011**
Microvascular invasion (yes vs. no)	1.5(1.0–2.2)	0.069	1.6(1.1–2.4)	**0.008**
Liver cirrhosis (yes vs. no)	2.1(1.0–4.4)	**0.039**	1.5(0.8–2.6)	0.206
BCLC stage (B/C vs. 0/A)	1.2(0.8–1.8)	0.417	1.2(0.9–1.8)	0.235
*Multivariate analysis*				
Tumorous QSOX1 (high vs. low)	0.6(0.4–0.9)	**0.018**	0.5(0.4–0.7)	**<0.001**
HBsAg (positive vs. negative)	6.0(1.5–24.8)	**0.013**	1.8(0.8–3.9)	0.145
Tumor encapsulation (no vs. yes)	n.a.		1.3(0.9–1.9)	0.126
Tumor differentiation (I–II vs. III–IV)	n.a.		1.5(1.0–2.2)	**0.041**
Microvascular invasion (yes vs. no)	n.a.		1.3(0.9–1.9)	0.238
Liver cirrhosis (yes vs. no)	1.5(0.7–3.2)	0.258	n.a.	

For tumorous QSOX1 median values were used as cutoff-point for definition of subgroups (low expression and high expression groups). Univariate analysis, Cox proportional hazards regression; Multivariate analysis, Cox proportional hazards regression; Variables were adopted in multivariate analysis for their prognostic significance by univariate analysis

*95% CI* 95% confidence interval, *n.a.* not applicable

The meaning of the bold *P* values is that they are under 0.05 and statistically significant

To validate the association of serum cf-QSOX1 with tumor QSOX1, the correlation between serum cf-QSOX1 levels and tumor QSOX1 densities in 12 patients was analyzed using paired serum and tumor tissues samples. A significant positive correlation between the values was found (Spearman’s rho = 0.643, *p* = 0.024).

Previous reports have suggested that QSOX1-S may function as a secreted oxidase and is present in most bodily fluids, whereas QSOX1-L is a transmembrane protein localized primarily in the Golgi or ER apparatus^25,26^. In the present study, two patterns of immunostaining for QSOX1 were observed, concentrated perinuclear cytoplasm (type I) and homogenous cytoplasm staining (type II). The perinuclear distribution is suggestive of Golgi or ER staining and thought to represent QSOX1-L localization, whereas homogenous cytoplasmic staining of QSOX1 may reflect the localization of QSOX1-S. However, specific antibodies against QSOX1 isoform are not commercially available. To test this hypothesis, Western blot was performed to determine expression levels of the two QSOX1 isoforms in 12 HCC tissues showing typical type I or type II immunostaining. As shown in Supplemental Fig. S8A, QSOX1 from the type I HCC tissues was found to be predominately at 83 kDa, whereas QSOX1 from the type II HCC tissues was shown to be almost entirely at 67 kDa, which suggests that perinuclear cytoplasmic staining represents the subcellular localization of the QSOX1-L isoform, and homogenous cytoplasm staining shows the primary subcellular localization of the QSOX1-S isoform. Given that serum cf-QSOX1 was found to be comprised of the QSOX1-S isoform, and was found to be significantly correlated with TTR in HCC patients, we attempted to analyze a potential association of QSOX1-S in tumor tissues with TTR and OS of HCC patients in the subgroup only with type II QSOX1 immunostaining (*n* = 105) from cohort B. As shown in Supplemental Fig. S8B, the QSOX1 density in this subgroup of type II staining was also found to be significantly associated with OS and TTR in HCC patients. Moreover, QSOX1 expression in the subgroup showing type II was more powerful as a prognostic marker than was the expression of QSOX1 in the entire cohort B (Fig. 1g, h). These results suggest that patients with higher QSOX1-S levels in their tumor tissues had a significantly better prognosis than did patients with low QSOX1-S levels. This observation supports the general results seen from the serum analysis.

### QSOX1-S inhibits HCC invasion and metastasis

**Fig. 2:**
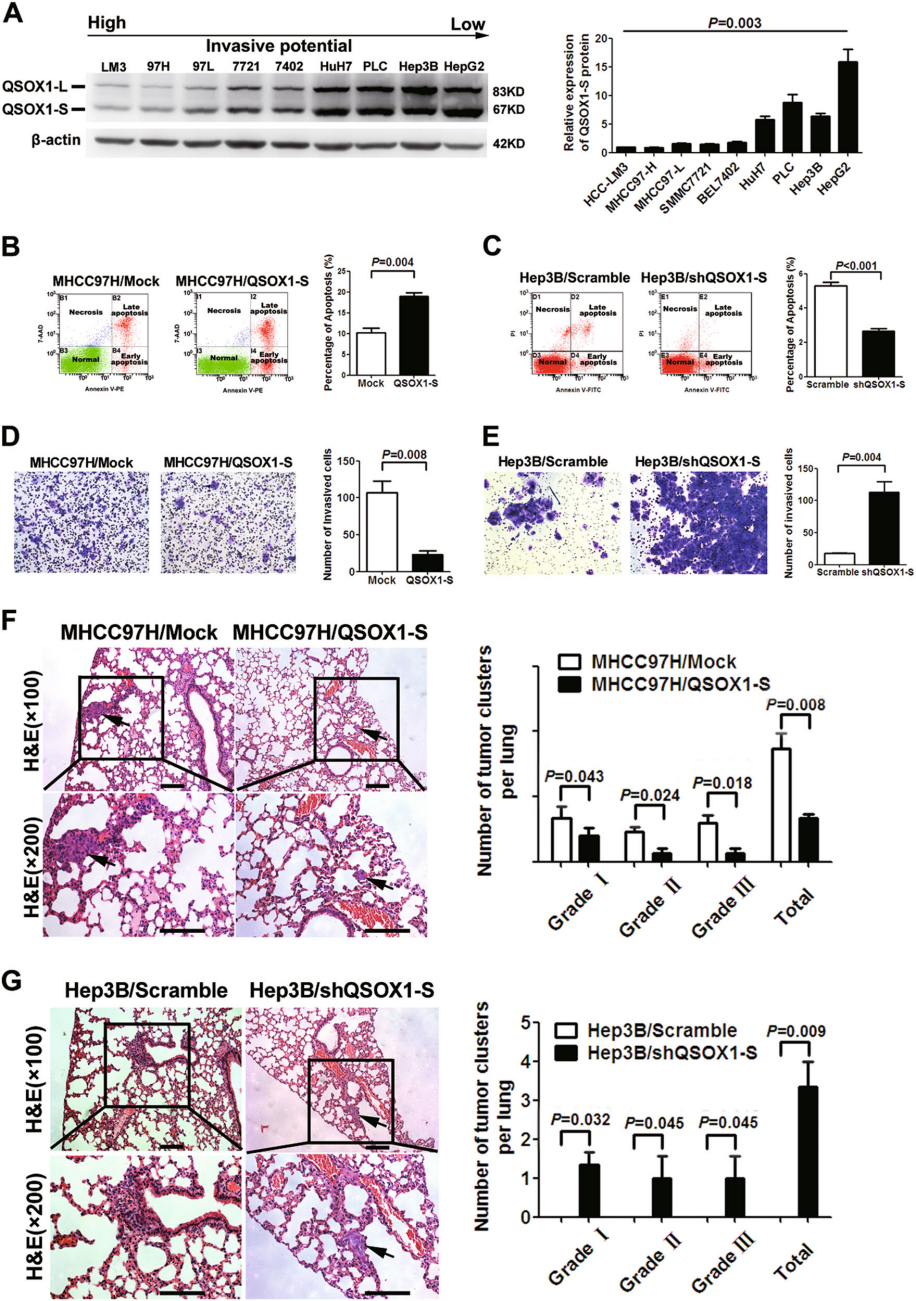
The effects of QSOX1-S on apoptosis and invasion of HCC cells in vitro and in vivo. **a** QSOX1 expression levels in HCC cell lines with different metastatic potentials were compared by Western blot. Relative quantification of the QSOX1-S expression levels is shown in right panel. Multiple comparisons were analyzed by one-way analysis of variance. **b** QSOX1-S overexpression increased the apoptosis of MHCC97-H cells (*n* = 6). **c** QSOX1-S knockdown inhibited apoptosis of Hep3B cells (*n* = 6). **d** The number of invaded cells in the MHCC97-H cells with QSOX1-S overexpression was significantly decreased as compared with controls (*n* = 6). **e** The number of Hep3B invaded cells with QSOX1-S knockdown were significantly increased as compared with controls (*n* = 6). **f** Metastatic lesions in the lungs of the orthotopic implantation models at 6 weeks after implantation are shown. The QSOX1-S overexpression resulted in decreased numbers and grade of lung metastatic lesions of MHCC97H cells in vivo (*n* = 8). Scale bar: 200 µm. **g** QSOX1-S knockdown led to increased incidence of lung metastasis of Hep3B cells in vivo (*n* = 8). The incidence of lung metastasis in control group (Hep3B/scramble) was 0%, thus numbers of lung metastatic lesions of Hep3B/scramble cells in vivo were 0. Scale bar: 200 µm. Values were expressed as the mean ± SD

We then examined whether expression levels of QSOX1-S in HCC cell lines correlated with metastatic potential. As shown in Fig. 2a, QSOX1-S levels were higher in those lines that showed a lower invasive and metastatic potential. We then explored the potential biologic function of QSOX1-S in HCC using an overexpression system and lentivirus-mediated knockdown (Supplementary Fig. S9). Figure 2b shows that an increased expression of QSOX1-S in the MHCC97-H cell line, which normally shows low endogenous QSOX1-S expression levels and a high metastatic potential, led to induced cell apoptosis. By contrast, QSOX1-S knockdown in Hep3B cells, which normally show low metastatic potential and high endogenous QSOX1-S expression, resulted in reduced cell apoptosis (Fig. 2c), increased cell proliferation compared with controls (Supplementary Fig. S10A). However, QSOX1-S overexpression in the MHCC97-H cells did not affect the proliferation of the cells (Supplementary Fig. S10B). We further assessed the impact of QSOX1-S on the invasive capacity of cells using a Transwell chamber assays. As shown in Fig. 2d, e, overexpression of QSOX1-S led to reduced MHCC97-H cell invasion, whereas knockdown of QSOX1-S had the opposite effect on Hep3B cells. These data support the observation that QSOX1-S is able to suppress invasion and promote apoptosis of HCC cells in vitro.

To further verify the role of QSOX1-S in HCC tumor growth and metastasis in vivo, orthotopic implantation models were established using the MHCC97-H and Hep3B cells that had been stably engineered for QSOX1-S-overexpression or QSOX1-S knockdown using shRNA lentiviruses. The incidence of lung metastasis of the MHCC97-H cells in orthotopic models in the QSOX1-S overexpression group was remarkably decreased as compared with the control group (25% vs. 75%). The total number and grade of lung metastatic lesions in the QSOX1-S overexpression group was also significantly lower than that seen in the control (Mock group) (Fig. 2f). By contrast, Hep3B orthotopic implantation models using the QSOX1-S-shRNA modified group showed a remarkably higher incidence of lung metastasis than did a scrambled control RNA group (37.5% vs. 0%) (Fig. 2g). In addition, QSOX1-S-shRNA transfection was found to significantly accelerate tumor growth in the Hep3B orthotopic models (Supplementary Fig. S10C). By contrast, QSOX1-S overexpression did not affect the tumor volume of the MHCC97-H models (Supplementary Fig. S10D). These data suggest that QSOX1-S acts as a metastatic suppressor in HCC, which is consistent with the finding that increased QSOX1-S is associated with long TTR and a better prognosis in HCC patients.

### The core-fucosylated glycan at Asn-130 is required for inhibitory effects of QSOX1-S on HCC invasion and metastasis

**Fig. 3:**
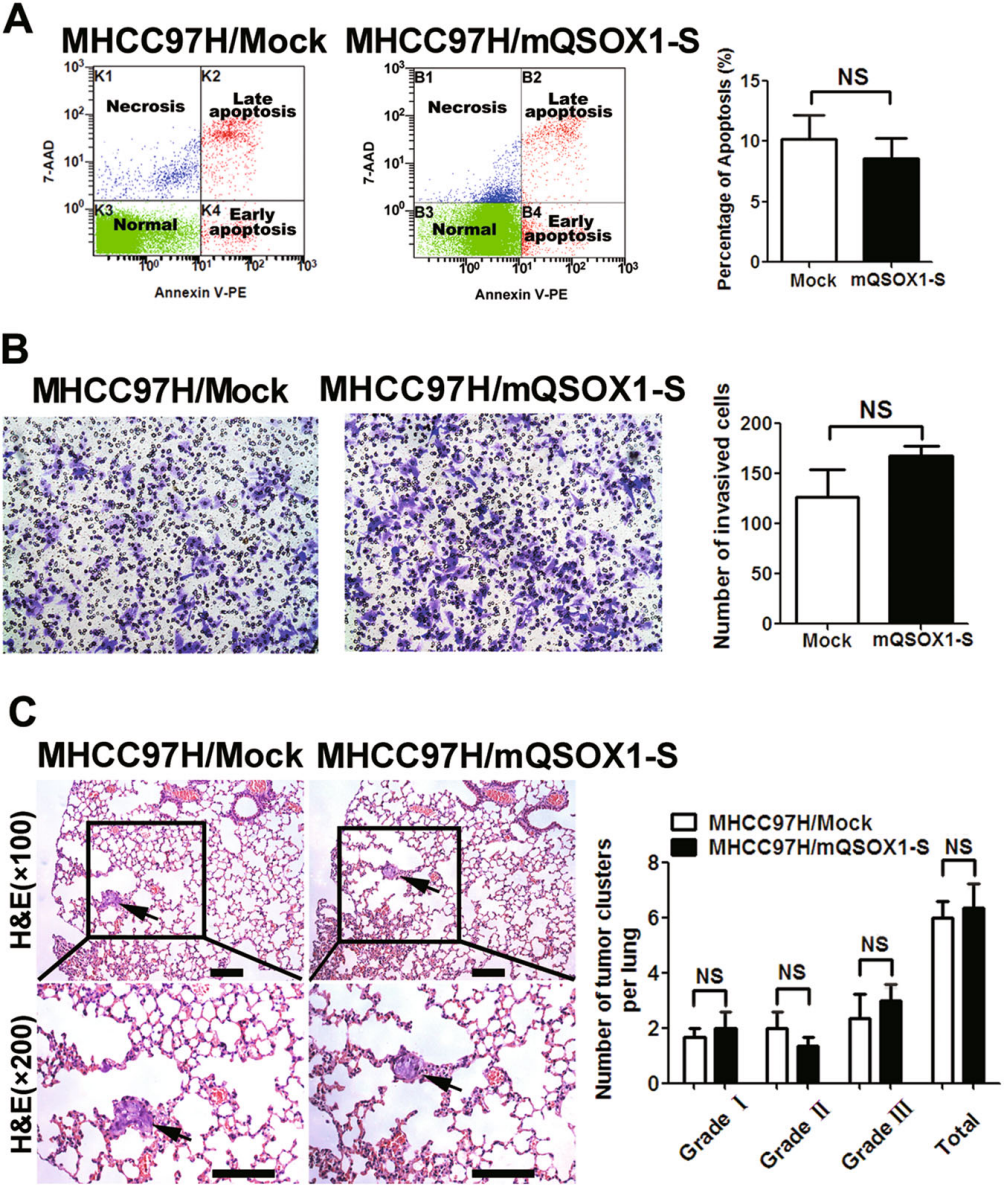
The effects of mutant QSOX1-S (mQSOx1-S) on apoptosis and metastasis of HCC cells. **a** The apoptosis levels of MHCC97H cells did not show significant differences between the mQSOX1-S overexpression group and control group (*n* = 6). **b** No significant difference in the number of invaded cells was seen between the mQSOX1-S overexpressed and control group in vitro (*n* = 6). **c** Metastatic lesions in the lungs of the orthotopic implantation models at 6 weeks after implantation are shown. The mQSOX1-S overexpression had no effect on lung metastasis of MHCC97H cells (*n* = 8). Scale bar: 200 µm

The mass spectrometric analysis showed that serum LCA glycopeptide from QSOX1 was glycosylated at the asparagine (Asn)-130 residue of QSOX1 (Supplemental Table S2), indicating that serum cf-QSOX1 is an N-linked glycoprotein with core-fucosylation at Asn-130. The core fucose refers to fucose attachment at the innermost reducing terminal glycan^27^. To investigate the biologic effect of core-fucosylated glycan at Asn-130 on QSOX1-S action, an MHCC97-H cell line was established stably overexpressing a mutated version of QSOX1-S (mQSOX1-S) with a mutation in the core-fucosylated glycan-binding region at Asn-130 (Supplementary Fig. S11). mQSOX1-S overexpression did not result in increased apoptosis of the MHCC97-H cells as was seen with QSOX1-S overexpression (Fig. 3a). In addition, mQSOX1-S overexpression did not influence the invasive capacity of MHCC97-H cells in vitro (Fig. 3b). These results suggest that the core-fucosylated glycan at Asn-130 is required for the effect of QSOX1-S on invasion and apoptosis of HCC cells.

We next examined the effects of mQSOX1-S overexpression on HCC metastasis in vivo using HCC orthotopic implantation models. As shown in Fig. 3c, no significant difference was found in the total number and grade of lung metastatic lesions between the mQSOX1-S overexpression group, and the controls. The incidence of lung metastasis was not significantly altered in the MHCC97-H orthotopic models when comparing the mQSOX1-S overexpression group to the control group (87.5% vs. 75%). These results support the observation that core fucosylated glycan at Asn-130 is necessary for an inhibitory effect of QSOX1-S on HCC metastasis.

### QSOX1 inhibits integrinβ1/FAK and EGFR/Raf/ERK signaling pathways

**Fig. 4:**
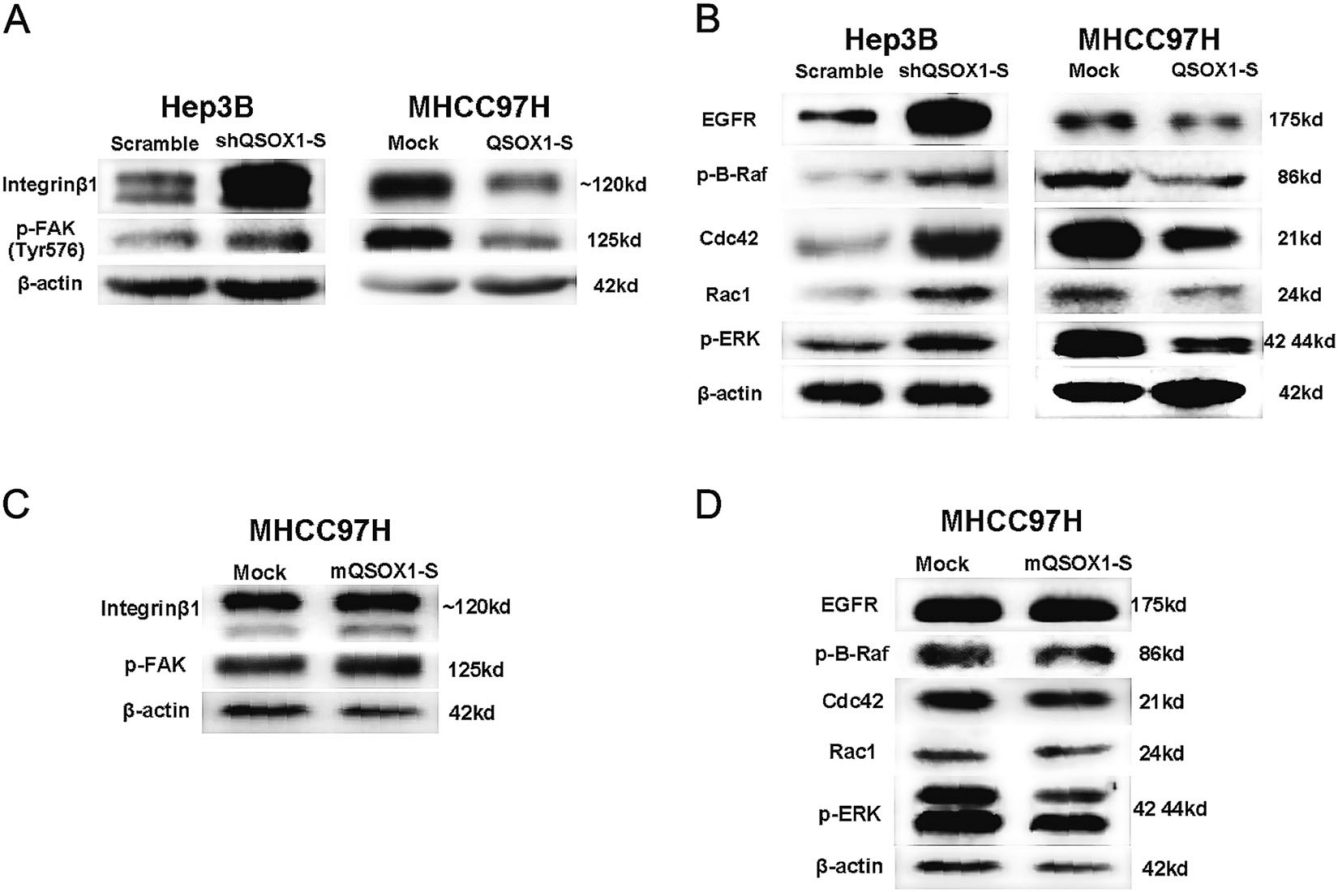
QSOX1-S inhibits integrinβ1/FAK and EGFR/Raf/ERK signaling pathways and its glycosylation at Asn-130 is necessary for this effect. Integrinβ1/FAK pathway-related molecules (**a**) and EGFR/Raf/ERK pathway-related molecules (**b**) in MHCC97-H cells with QSOX1-S overexpression and Hep3B cells with QSOX1-S knockdown were analyzed by western blotting. Likewise, western blot analyses of integrinβ1/FAK pathway-related molecules (**c**) and EGFR/Raf/ERK pathway-related molecules (**d**) in MHCC97-H cells with mQSOX1-S overexpression are shown

In view of inhibitory effects of QSOX1-S on HCC, we sought to verify whether QSOX1-S would influence two important signaling pathways linked to HCC biology, namely; integrinβ1/FAK and EGFR/Raf/ERK. The protein levels of different members of the signaling pathway were then evaluated in the HCC cell lines with either QSOX1-S overexpression, or knockdown. As shown in Fig. 4a, knockdown of QSOX1-S in Hep3B increased integrinβ1 and phosphorylated FAK levels, whereas ectopic expression of QSOX1-S in MHCC97-H cells led to a significant decrease in integrinβ1 and phosphorylated FAK. In addition, knockdown of QSOX1-S in Hep3B led to an increase in EGFR, phosphorylated B-Raf, phosphorylated ERK, Cdc42, and Rac1, whereas ectopic expression of QSOX1-S in MHCC97-H resulted in a decreased expression of these molecules (Fig. 4b). These results suggest that QSOX1-S can inhibit the integrinβ1/FAK and EGFR/Raf/ERK signaling pathways in HCC.

In addition, we found that overexpression of mQSOX1-S in MHCC97-H did not influence integrinβ1, phosphorylated FAK, EGFR, phosphorylated B-Raf, phosphorylated ERK, Cdc42, or Rac1 expression as was seen with QSOX1-S overexpression (Fig. 4c, d). These results suggest that core-fucosylated glycan at Asn-130 is required for the inhibiting effect of QSOX1-S on the integrinβ1/FAK and EGFR/Raf/ERK signaling pathways.

## Discussion

QSOX1 is a sulfhydryl oxidase that is thought to participate in redox reactions during intracellular protein folding^28–30^. In the present study, we show that high serum cf-QSOX1 correlates with long TTR of patients after HCC resection, and that QSOX1 in tumor tissues is associated with a good prognosis in HCC patients. Univariate and multivariate analyses was able to demonstrate that both serum cf-QSOX1, and tumor expressed QSOX1, act as independent prognostic factors. Serum levels of cf-QSOX1 were found to be associated with disease outcome in patients with early-stage HCC. Importantly, current clinical staging systems generally fail to provide an accurate prognostic assessment for these patients. The results presented here suggest that serum cf-QSOX1 levels may be useful for determining clinical outcome, especially in those patients with early stage HCC. Further validation of this hypothesis using larger cohorts represents an important next step to demonstrate how robust the analysis is in diverse clinical settings. Our data further indicate that serum cf-QSOX1 is made up of QSOX1-S, and is closely related to tumor levels of QSOX1. It is possible that serum cf-QSOX1 derives largely from HCC tissues where the tumor cells produce cf-QSOX1-S, which is then able to enter the bloodstream. But this remains to be confirmed. Serum cf-QSOX1-S may represent an important new biomarker for metastatic recurrence of HCC.

We were able to functionally demonstrate via in vitro and in vivo experiments that QSOX1-S can have a direct inhibitory effect on the invasive and metastatic potential of HCC. QSOX1 has been found to be over-expressed in multiple types of cancers, however, controversy exists regarding the action of QSOX1 as a tumor suppressor or promoter^31–33^. In the study presented here, the sample analysis and functional investigation all support the idea that QSOX1-S acts as a suppressor of metastases in HCC. However, it is possible that QSOX1-S may play disparate roles in differential tumor types depending on the given context.

Gain-of function and loss-of function studies of QSOX1-S that were directed towards determining general effects on the proliferation of HCC cells and tumor growth, yielded inconsistent results. This may be due to the fact that QSOX1-S and QSOX1-L are largely composed of identical sequences^23^, and a knockdown of QSOX1-S may have also reduced expression of QSOX1-L. An inhibitory effect may require the combined action of QSOX1-S and QSOX1-L—an issue that remains to be determined.

QSOX1 was reported to contain three potential N-glycosylation sites, Asn-130, Asn-243, and Asn-575^34,35^. We show here that serum QSOX1 from HCC patients have a core-fucosylated glycan at the Asn-130 residue. Importantly, we found that the inhibitory effect of QSOX1-S on invasion, and metastasis of HCC, was dependent on core-fucosylated glycan at Asn-130, suggesting vital roles for core-fucosylated glycan in maintaining biological functions of QSOX1-S in HCC progression. Core-fucosylation of proteins has been reported to be involved in many tumor processes, such as HCC, pancreatic cancer, lung cancer and ovarian cancer^36–39^. In our study, mutant QSOX1 showed a glycan depletion at Asn-130 residue, instead of only a depleted core-fucose attached on glycan, therefore, whether core-fucose plays an essential role in inhibitory effect of QSOX1-S on metastasis of HCC remains to be demonstrated.

Several studies have suggested that QSOX1 is likely important in ECM formation/remodeling^23,40^. Integrin/FAK pathway signaling mediates the adhesion of cells to the ECM which is essential for the invasion and metastasis of tumor cells^41^. Given our findings that QSOX1-S suppresses integrin/FAK and EGFR/Raf/ERK pathways, we propose that QSOX1-S may in part also suppress tumor invasion and metastasis, and promote apoptosis of HCC cells through an inhibition of the integrin/FAK and EGFR/Raf/ERK pathways. Interestingly, the inhibitory action of QSOX1-S on integrin/FAK and EGFR/Raf/ERK pathways also requires a core-fucosylated glycan on Asn-130 of QSOX1. Since integrinβ1, ERK, and EGFR all harbor sulfhydryl groups on their peptide backbones, it is possible that QSOX1 may affect the function of these molecules by catalyzing their sulfhydryl groups to form disulfide bonds, and therefore altering their tertiary or quaternary structures.

In summary, serum cf-QSOX1-S and tumor QSOX1 are potential biomarkers for predicting clinical outcome in HCC patients. The cf-QSOX1-S is a suppressor of invasion and metastasis of HCC, and providing exogenous cf-QSOX1-S may be a helpful strategy to control tumor relapse and further improve the therapeutic effect of HCC patients.

## Materials and methods

### Patients and samples

A total of 321 HCC patients who had undergone curative resection (in Shanghai, China) from January 2003 to December 2010 were enrolled in the study. The diagnosis of HCC was confirmed by two pathologists. Curative resection for HCC is defined as complete resection of the tumor when the margins are free of cancer by histological examination. All patients were assigned into two cohorts. Cohort A (*n* = 140) was used to identify serum recurrence-associated glycoproteins. The specimens from this cohort included 40 tumor tissues samples (set A) and 100 serum samples (sets B and C). Cohort B (*n* = 193) was used to validate the prognostic values of the glycoproteins identified in the HCC patients. The specimens from cohort B consisted of 193 tumor tissue samples. A total of 12 patients in this study had paired serum (from set C) with tumor tissue samples (from cohort B). The clinicopathological characteristics are summarized in Supplemental Table S5. The serum samples were collected on third day before hepatectomy for HCC, and the tissue specimens were formalin-fixed paraffin-embedded (FFPE).

### Cell lines and cell culture

Fourteen cell lines including HCC-LM3, MHCC97L, MHCC97H, SMMC7721, SMMC-7402, HuH7, PLC, Hep3B, HepG2, and cell lines derived from MHCC97H and Hep3B were used. Their source were described in our previous study^42^.

### Lectin affinity chromatography of serum samples

The experiments were performed as previously described^20^. Briefly, the serum samples were digested with trypsin at an enzyme-to-substrate ratio of 1:50 (w/w) at 37 °C for 16 h. The digested serum samples were subjected to lectin affinity chromatography for separating the glycopeptides with specific glycan structure using ConA-based, LCH-based, and WGA-based isolation kits.

### Tissue microarray and immunohistochemistry

Tissue microarrays were constructed and immunohistochemistry (IHC) was performed as described previously^21^. The density of positive staining was evaluated using a Leica CCD camera DFC420 connected to a Leica DM IRE2 microscope (Leica Microsystems Imaging Solutions Ltd., Cambridge, UK). The QSOX1 densities were counted by Image-Pro Plus v5.0 software (Media Cybernetics Inc., Bethesda, MD, USA)^21^. A uniform setting was applied for QSOX1 antibody staining. The integrated optical density (IOD) of all positive staining in each photograph was measured; the mean QSOX1 density was calculated as the product of IOD/total area.

### Plasmid construction, transfection, lentiviral production, infection, and establishment of stable cell lines

Detailed methods can be found in the Supplementary files. Primer sequences were shown in Supplemental Table S6.

### Cell invasion assay

The invasive ability of HCC cells was determined using 24-well transwell chambers (Costar, Cambridge, MA, USA) coated with Matrigel (BD Pharmingen, San Jose, CA, USA) as described in previous study^42^. Briefly, 5 × 10^4^ cells in serum-free medium were seeded on a membrane (8.0-μm pores) in a 24-well plate. Medium containing 10% fetal bovine serum was added to the lower chamber of each well. After 48 h incubation, cells that migrated to the underside of the membrane were stained with Giemsa (Sigma Chemical Company, Saint Louis, MO), imaged, and counted with a microscope (Leica, UK) at ×200 magnification. All experiments were performed in triplicate.

### Nude mouse model

All experimental procedures involving animals were approved by The Animal Care and Use Committee of Fudan University, China. Flank xenografts were established by subcutaneous implantation of 2 × 10^6^ HCC cells into nude mice (BALB/c nu/nu, 4 weeks old) (SLRC, Shanghai, China). Subcutaneous tumors were removed and dissected into 1-mm^3^ sections, which were implanted into the left hepatic lobe of BALB/c nude mice to establish orthotopic implantation models. Tumor growth was monitored, and the mice were sacrificed at 6 weeks. The tumors, livers, and lungs were removed, fixed in formalin, and embedded in paraffin. Consecutive sections were made for every lung tissue block and stained with hematoxylin and eosin. The number of lung metastases was calculated and evaluated independently by two pathologists. Based on the number of HCC cells in the maximal section of the metastatic lesion, lung metastases were classified into four grades. Grade I was defined as having ≤20 tumor cells; grade II had 20–50 tumor cells; grade III had 50–100 tumor cells; grade IV had >100 tumor cells.

### Statistical analysis

Statistical analysis was performed using SPSS version 15.0 (SPSS Inc., Chicago, IL, USA). Comparison of two groups was conducted using the Student *t*-test. Multiple comparisons were analyzed by one-way analysis of variance. The log-rank (Mantel–Cox) test was used for patient survival and recurrence analysis. Univariate and multivariate analyses were based on the Cox proportional hazards regression model. For all immunohistochemical variables, the median value (0.01064) of QSOX1 density (IOD) was used as the cutoff for defining patient subgroups of high or low QSOX1 expression. Two-tailed *p*-values < 0.05 were considered significant.

Additional details are provided in the supplement.

Additional details are provided in supplementary files.

## Supplementary information

revised-supplemental materal
