# Supplementary material for: Core fucosylated glycan-dependent inhibitory effect of QSOX1-S on invasion and metastasis of hepatocellular carcinoma
Source: Cell Death Discov. 2019 Apr 3;5:84. doi: 10.1038/s41420-019-0164-8 (PMC6447561; doi:10.1038/s41420-019-0164-8)
Supplement: Supplementary file 1 — revised-supplemental materal [file 41420_2019_164_MOESM1_ESM.pdf]

Supplementary Materials and Methods

Antibodies and reagents

The following antibodies and reagents were used in this study: Control β-actin antibody (1:2000) and Integrinβ1 antibody (1:2000) were purchased from Abcam (Cambridge, UK,). QSOX1 antibody (1:2000) was obtained from Millipore (USA). Antibodies to phospho-FAK (1:2000), phospho-ERK (1:2000), EGFR (1:2000), phospho-B-Raf (1:2000), Cdc42 (1:2000), Rac1 (1:2000) were purchased from Cell Signaling Technology Inc (Beverly, MA). Antibodys to GAPDH (1:2000, Cell Signaling Technology, Danvers, MA), Goat anti-Mouse and Goat anti-Rabbit HRP Conjugate (1:5000) were purchased from Cwbiotech (Shanghai, China). Protein was detected with Image Acquisition using ImageQuant™ LAS 4000 (GE Healthcare Life Sciences, Michigan, USA).

Patients and samples

All patients didn't receive any preoperative anticancer treatment. Their follow-up ranged from 2 to 110.2 months. Follow-up assays included serum alpha-fetoprotein (AFP), ultrasonography, and chest X-ray every 2–3 months after surgery. Computed tomography (CT) or magnetic resonance imaging (MRI) was done when recurrence was suspected. A diagnosis of recurrence was based on typical appearance on CT and/or MRI. A further treatment was given if tumor recurrence was diagnosed according to a uniform guideline as previously described [1, 2].This study was approved by the Fudan Research Ethics Committee. Informed consent was obtained

according to the committee’s regulations and the Declaration of Helsinki.

Cell lines and cell culture

All cell lines were routinely cultured in Dulbecco’s modified Eagle’s medium (DMEM) (Gibco BRL, Grand Island, USA) supplemented with 10% (v/v) fetal bovine serum (FBS) (Gibco BRL) at 37°C in a humidified incubator containing 5% CO2.

Preparation of serum samples

The serum samples were stored at −80°C before processing. The most abundant serum proteins were removed by the ProteoMiner Protein Enrichment Kit according to the manufacturer's instruction. The protein concentrations were determined using the BCA assay kit.

Lectin microarrays

The patients of set A (n=40) were divided into recurrence group (n=20) and non-recurrence group (n=20). Every 5 samples from the same group were equally mixed, and applied to Lectin microarrays which had been developed in our institute. Lectin microarrays were performed as previously described [3]. Briefly, proteins in tissue samples were labeled with Cy5 fluorescent dye (Piscataway, USA), and then the Labeled glycoproteins were separated from the excess free dye by gel filtration chromatography (Sephadex G-50). After the microarray slides were blocked, Cy5-labeled glycoproteins were applied to separate wells on slides and incubated at 30°C for 2 h. Finally, the slides were scanned with Lux-Scan 10K-A Scanner (PMT = 750).

Isotope labeling with ^{18}O or ^{16}O water

After lectin affinity chromatography, the glycoproteins obtained from the samples were desalted using SepPak C18 cartridges, and then dried in a vacuum centrifuge. The glycoproteins were then mixed with immobilized trypsin (20% slurry v/w) for 20 min with gentle shaking, and then lyophilized. The lyophilized peptides were dissolved in 100 μL acetonitrile in 50 mM NH4HCO3 (pH 6.8) (ACN/NH4HCO3, 20% v/v) prepared with $H_2{}^{16}O$ or $H_2{}^{18}O$ in advance, then incubated at 37°C for 24 h to catalyze the labeling of tryptic peptides at the C-terminus. The immobilized trypsin beads were then removed by MicroSpin columns. A total of 5 μL formic acid was added to further inhibit any possible residual trypsin activity. The peptides were lyophilized and then dissolved in 100 mM NH4HCO3 buffer prepared in $H_2{}^{16}O$ or $H_2{}^{18}O$. PNGase F was added at a concentration of 1 μL PNGase-F/mg of crude protein, and the labeling was conducted at 37°C overnight. Finally, the ^{16}O- and ^{18}O-labeled peptides were mixed at a ratio of 1:1 for samples from recurrence and non-recurrence group and lyophilized.

Nano LC-electrospray ionization (ESI)-MS/MS

The lyophilized peptides were resuspended with 2% ACN in 0.1% formic acid, separated by nano LC, and then analyzed by online electrospray tandem mass spectrometry. The experiments were performed on a Nano Aquity UPLC system (Waters) connected to an LTQ Orbitrap XL mass spectrometer (Thermo Electron Corp., Bremen, Germany) interfaced with an online nano electrospray ion source (Michrom Bioresources, Auburn, CA). The peptide separation was performed in a Michrom CAPTRAP (500 μm i.d. × 2 mm trap column) and a Michrom C18 (3.5 μm, 100 μm i.d. × 15 cm reverse phase column) (Michrom Bioresources). The model

glycoprotein digests (0.5 μg) were loaded onto the trap column and leached at a flow rate of 20 μL/min for 3 min. The mobile phases included 2% ACN in 0.1% formic acid (phase A and the loading phase) and 95% ACN in 0.1% formic acid (phase B). To achieve sufficient separation, a 60-min (for glycoprotein standards) or 90-min (for serum samples) linear gradient from 5% to 45% at phase B was employed. The flow rate of the mobile phase was set at 500 nL/min, and the electrospray voltage used was 1.6 kV. The linear gradient was adjusted to 90 min for serum samples analyses, while all other parameters remained unchanged. The LTQ Orbitrap XL mass spectrometer was operated in the data-dependent mode with an automatic switch between MS and MS/MS acquisition. The survey full-scan MS spectra with two microscans (m/z 350–1800) was acquired in Orbitrap at a resolution of 100,000 (at m/z 400) followed by eight MS/MS scans in LTQ trap. Dynamic exclusion was set to initiate a 60 s exclusion for ions analyzed twice within a 10 s interval.

Western blot

Western blot was conducted as described previously [2]. Briefly, the tested cells were subjected to lysis with RIPA buffer, and the cell lysates were separated by electrophoresis and transferred to PVDF membrane. After blocking, the membranes were incubated with primary antibodies, and subsequently incubated with horseradish peroxidase-conjugated secondary antibody. The signal was detected by exposure to X-ray film.

Plasmid construction, transfection, lentiviral production, infection, and establishment of stable cell lines

Human lentiviral shRNA against human QSOX1 was designed. pLKO.1-TRC cloning vector (Sigma-Aldrich, USA) was used. And pCDH-EF1-MCS-IRES-GFP vector (System Biosciences, USA) was used for QSOX1-S and mQSOX1-S overexpression. Primers (Sangon Biotech, Shanghai, China) for constructing plasmids are shown in Supplementary Table S6. The QSOX1-S, mQSOX1-S and QSOX1-shRNA oligos were purified, reannealed, and then cloned into lentiviral vectors. Lentiviruses were generated by transfecting lentiviral-expressing vectors pCDH-QSOX1-S, pLKO.1-shQSOX1, pCDH-GFP, pLKO.1-Sramble, together with packaging vector psPAX2 and envelope plasmid pMD2.G into 293T cells. At 48 to 72h after transfection, viral culture supernatants from 293T cells were collected and used to infect the exponentially growing target cells MHCC97H or Hep-3B with 50% confluence in the presence of polybrene (Sigma-Aldrich). Cells were passaged after 48 to 72 h of infection. Drug selection of infected cells was performed with puromycin (Invitrogen Life Technologies, Inc., Carlsbad, CA) to purify polyclonal-infected populations of QSOX1-shRNA, QSOX1-S or mQSOX1-S-expressing cells. The effectiveness of shRNA knockdown and overexpression was confirmed by Western blotting with specific antibody anti-QSOX1.

Cell Proliferation and Apoptosis Analyses

Cell proliferation was examined using Cell Counting Kit-8 (Dojindo, Kumamoto, Japan). According to the manufacturer’s instructions for Cell Counting Kit-8, harvested cells were seeded in 96-well plates at 1×103 per well in a final volume of 100 μL and then cultured for 24, 48, 72, and 96 hours. CCK-8 solution (10 μL) was

added into each well, and the absorbance at 450 nm was measured after incubation for 2 hours at 37°C to calculate the number of viable cells. Apoptosis analyses were performed using propidium iodide (Keygen, Nanjing, China) and phycoerythrin (PE)-annexin apoptosis detection kit (BD Pharmingen, San Jose, CA, USA) on flow cytometry (Epics Altra, Beckman Coulter， USA).

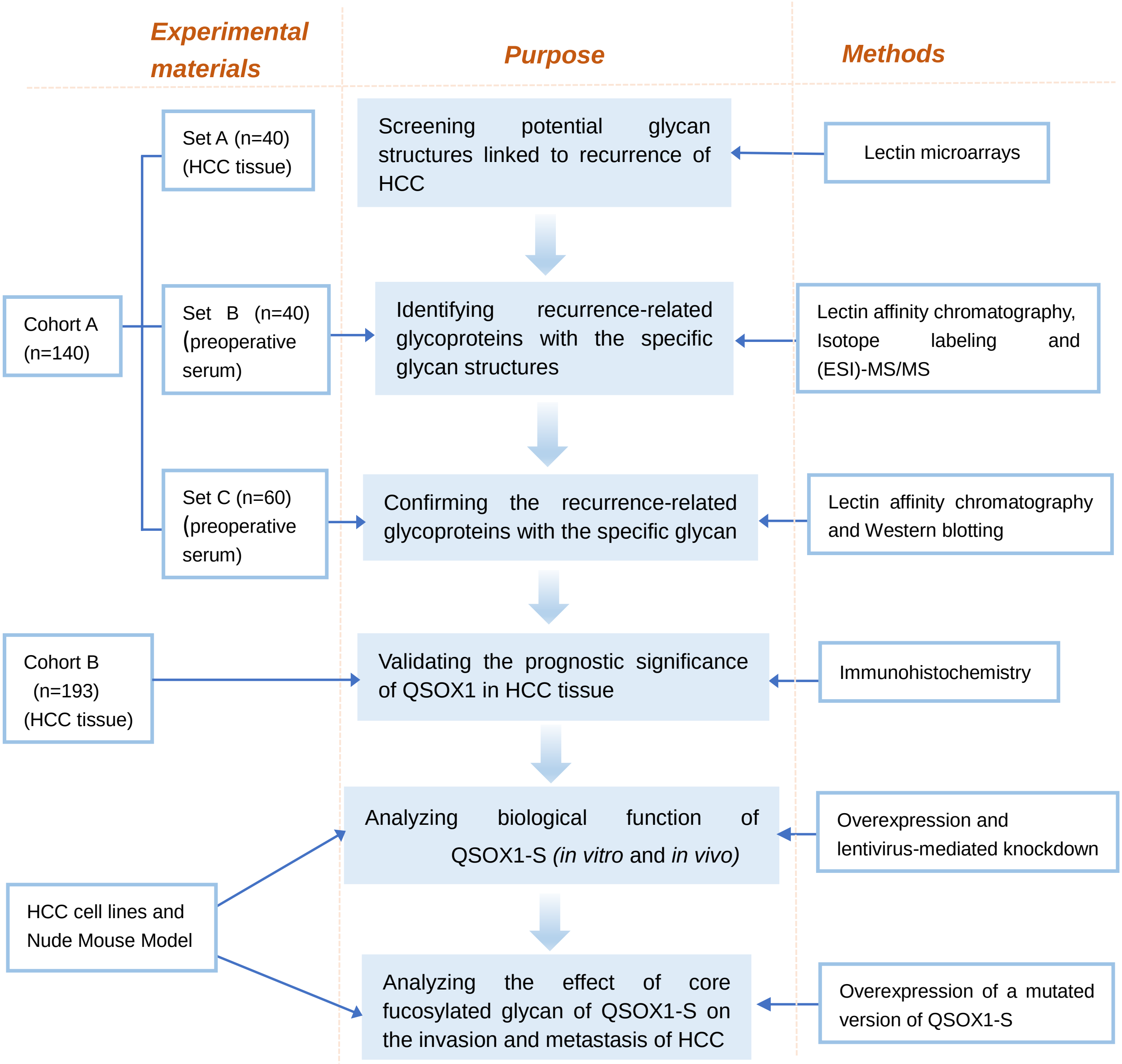

Supplemental Figure S1. The flowchart of this study.

A

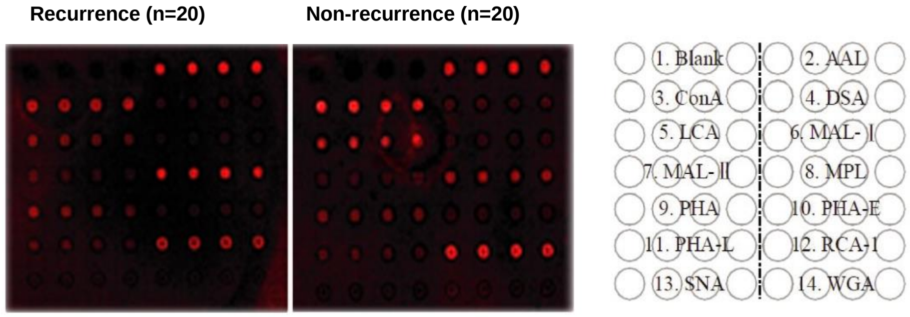

B

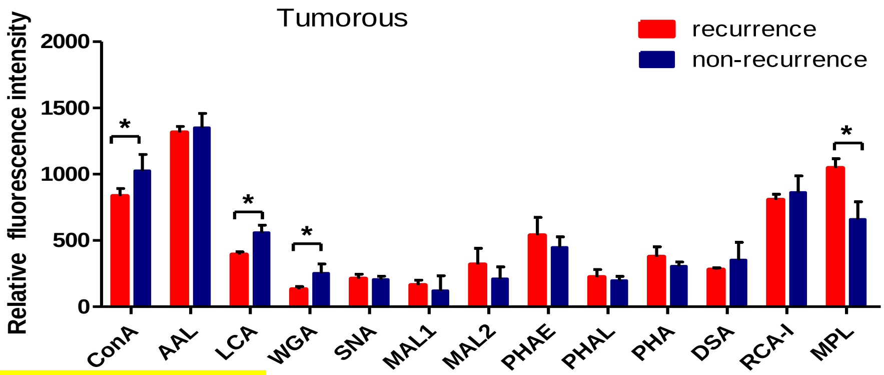

Supplemental Figure S2. Lectin microarrays analyses for glycan structure related to metastatic recurrence of HCC. (A) The lectin microarrays were scanned by fluorescence scanner after incubation with Cy5-labeled proteins of tumorous tissues from HCC patients of set A (n=40), and the representative scanned images were shown. Right panel is the distribution diagram of arrayed lectins on microarray. (B) Statistical analyses in A were performed between recurrence and non-recurrence groups.

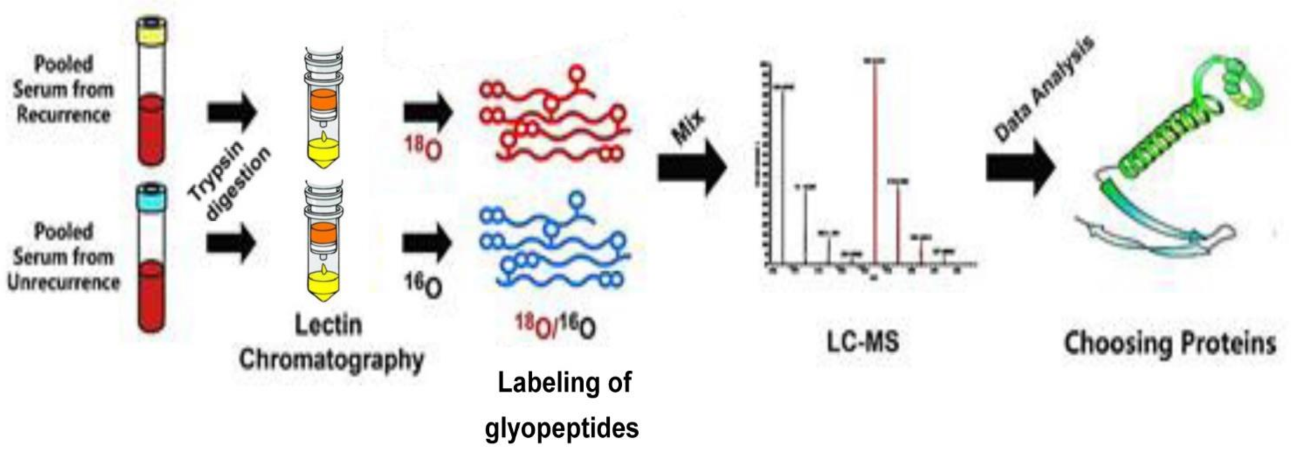

Supplemental Figure S3. Flow chart of identifying recurrence-related glycoprotein with specific glycan structure/type of glycosylation. Serum samples from recurrence group or non-recurrence group were pooled respectively. Glycoproteins from serum samples were digested into glycopeptides with trypsin. Then, glycopeptides were enriched via lectin affinity chromatography from two different groups of sample, desalted, and then catalyzed by immobilized trypsin and PNGase-F in ^{18}O or ^{16}O water. Equal amounts of ^{18}O- and ^{16}O-labeled glycopeptides were mixed, and the 6 Da mass shifts were generated between paired, labeled glycopeptides, which could be identified by subsequent LC-ESI-MS/MS.

Supplemental Table S1. Sammary of glycoproteins with abundance changes between patients with HCC recurrence (labeled ^{18}O) and those without HCC recurrence (labeled ^{16}O).

Lectin	*UniProtKB*	*Glycoprotein*	*Median*	*SD*
ConA	P00450	CERU_HUMAN	1.94	1.01
ConA	P10909	CLUS_HUMAN	1.66	0.62
ConA	P0C0L4	CO4A_HUMAN	2.05	0.67
ConA	P0C0L5	CO4B_HUMAN	2.11	0.72
ConA	P02748	CO9_HUMAN	1.49	0.50
ConA	O75636	FCN3_HUMAN	1.46	0.92
ConA	P02675	FIBB_HUMAN	1.63	0.86
ConA	P02679	FIBG_HUMAN	6.81	1.67
ConA	P01877	IGHA2_HUMAN	1.65	0.32
ConA	P01857	IGHG1_HUMAN	1.25	0.21
ConA	P01860	IGHG3_HUMAN	1.61	0.86
ConA	P01871	IGHM_HUMAN	8.90	4.71
ConA	P01591	IGJ_HUMAN	1.76	0.08
ConA	Q04759	KPCT_HUMAN	0.20	1.31
ConA	Q08380	LG3BP_HUMAN	1.72	0.15
ConA	Q9C099	LRCC1_HUMAN	0.28	0.01
ConA	P48740	MASP1_HUMAN	0.34	0.18
ConA	P04220	MUCB_HUMAN	8.68	5.28
ConA	Q96QU1	PCD15_HUMAN	0.32	0.13
ConA	Q9Y6X2	PIAS3_HUMAN	0.26	0.03
ConA	P27169	PON1_HUMAN	1.48	0.41
ConA	Q92954	PRG4_HUMAN	1.84	1.15
ConA	P00734	THRB_HUMAN	1.28	0.10
ConA	P04004	VTNC_HUMAN	2.09	0.10
LCA	P04114	APOB_HUMAN	2.60	0.06
LCA	P05090	APOD_HUMAN	1.74	0.05
LCA	P02745	C1QA_HUMAN	1.76	0.74
LCA	P00450	CERU_HUMAN	1.71	0.07
LCA	P10909	CLUS_HUMAN	0.81	0.03
LCA	P0C0L4	CO4A_HUMAN	1.39	0.10
LCA	P0C0L5	CO4B_HUMAN	1.37	0.11
LCA	P23142	FBLN1_HUMAN	5.77	0.50
LCA	P02675	FIBB_HUMAN	1.24	0.09
LCA	P02679	FIBG_HUMAN	0.47	0.05
LCA	P01871	IGHM_HUMAN	0.79	0.22
LCA	Q08380	LG3BP_HUMAN	0.69	0.01
LCA	Q9C099	LRCC1_HUMAN	0.32	0.03
LCA	A4QPB2	LRP5L_HUMAN	8.67	0.43
LCA	P04220	MUCB_HUMAN	0.53	2.62
LCA	Q96QU1	PCD15_HUMAN	0.20	0.01

LCA	P05164.1	PERM_HUMAN	0.23	0.10
LCA	P80108.3	PHLD_HUMAN	4.73	1.82
LCA	P55058.1	PLTP_HUMAN	3.20	1.40
LCA	O00391	QSOX1_HUMAN	0.56	0.51
LCA	P04004	VTNC_HUMAN	0.14	0.05
LCA	Q9C0J8	WDR33_HUMAN	5.69	0.11
WGA	Q13510	ASAH1_HUMAN	0.24	0.04
WGA	P00450	CERU_HUMAN	4.27	0.39
WGA	P10909	CLUS_HUMAN	1.27	0.10
WGA	P0C0L4	CO4A_HUMAN	2.78	0.29
WGA	P02675	FIBB_HUMAN	8.70	3.83
WGA	P02679	FIBG_HUMAN	3.14	0.34
WGA	Q9C099	LRCC1_HUMAN	0.30	0.02
WGA	Q5T2W1	NHRF3_HUMAN	0.47	1.37
WGA	Q96QU1	PCD15_HUMAN	0.50	0.04
WGA	O43815	STRN_HUMAN	2.36	1.17
WGA	P04004	VTNC_HUMAN	1.42	0.05

Supplemental Table S2. Mass spectrometry information about the glycoproteins selected to further confirm their association with HCC recurrence.

**UniProtKB**	**Glycoprotein**	**Glycopeptide Sequence**	**Lectin used for affinity**	**N-sites (aa)**
P00450	CERU_HUMAN	EGAIYPDN*TTDFQR	ConA	138
Q08380	LG3BP_HUMAN	ALGFEN*ATQALGR	LCA	69
O00391	QSOX1_HUMAN	N*GSGAVFPVAGADVQTLR	LCA	130

Note: aa, amino acid residue. *N-linked glycosylation site.

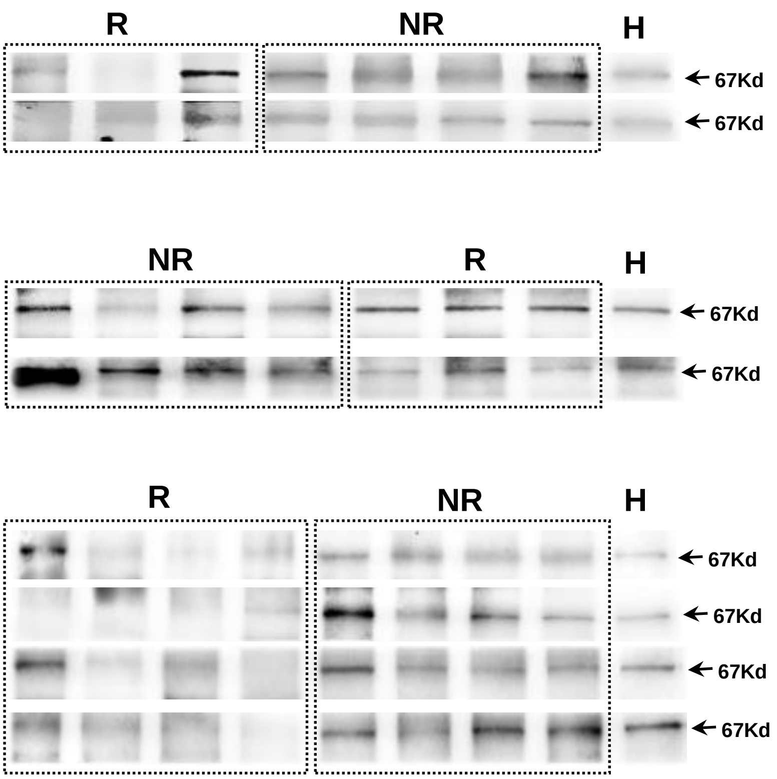

Supplemental Figure S4. The analyses of LCA-combinable QSOX1 (cf-QSOX1) expression levels in serum from 60 patients with HCC. The serum glycoproteins were enriched by LCA lectin affinity chromatography, and then followed by western blotting against QSOX1. The cf-QSOX1 bands were localized at 67Kd. The density of all cf-QSOX1 bands from HCC patients were normalized by band density of the same healthy individual. R denotes patients with HCC recurrence; NR denotes patients without HCC recurrence; H denotes same healthy individual.

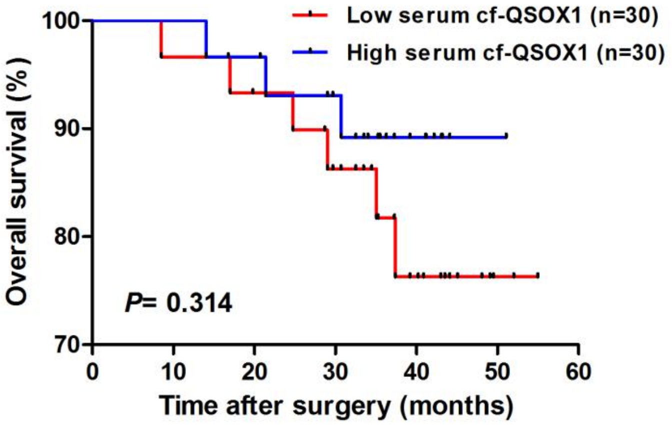

Supplemental Figure S5. Kaplan-Meier curves for overall survival (OS) in HCC patients of set C according to serum cf-QSOX1 level. The median QSOX1 densities were used as the cutoff for the definition of subgroups.

Supplemental Table S3. Statistics of the association of indicated glycoproteins with OS and TTR of patients.

Lectin used for affinity	Glycoprotein	*p* Value （OS）	*p* Value （TTR）
ConA	CERU	0.093	0.560
LCA	LG3BP	0.670	0.883

Note: The serum glycoproteins were enriched by ConA or LCA lectin affinity chromatography, and then followed by western bloting against indicated glycoproteins. Kaplan-Meier analyses for TTR and OS analyses were performed using the median indicated glycoproteins densities as the cutoff for the definition of subgroups. OS, Overall survival; TTR, Time to recurrence

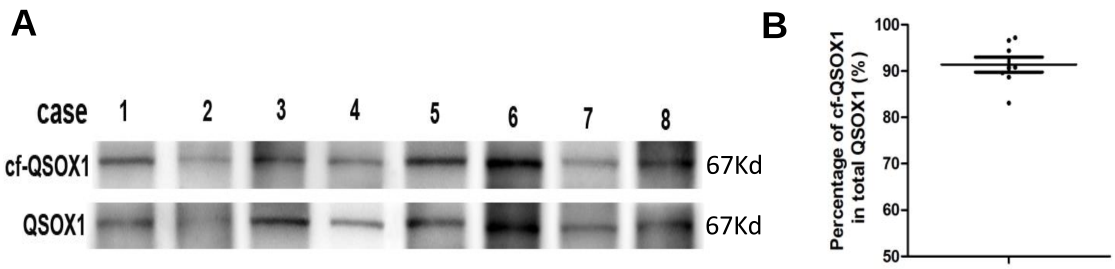

Supplemental Figure S6. Serum cf-QSOX1 accounts for a substantial percentage of serum total QSOX1. Serum samples from 8 patients (4 patients with HCC recurrence and 4 patients without HCC recurrence) were divided into two equal parts respectively. One was subjected to enrichment of glycoprotein by LCA lectin affinity chromatography, and then followed by western blotting against QSOX1. Another one was directly subjected to western blotting against QSOX1. (A) Representative western blot analysis was shown. (B) The percentages of cf-QSOX1 in total QSOX1 were calculated.

Supplemental Table S4. The association of tumorous QSOX1 density with clinicopathologic feature

Characteristic	tumorous QSOX1 *(n=193)* Low (n=96)	High (n=97)	p value
Sex			
Male	76	13	0.170
Female	20	84	
Age			
≤51 years	48	46	0.720
>51 years	48	51	
HBsAg			
Negative	7	10	0.460
Positive	89	87	
AFP			
≤20 ng/ml	31	32	0.918
>20 ng/ml	65	65	
ALT			
≤75 U/L	83	90	0.149
>20 U/L	13	7	
Tumor size			
≤3cm	42	34	0.216
>3cm	54	63	
Tumor number			
Single	94	88	0.058
Multi	2	9	
Microvascular invasion			
No	68	60	0.187
Yes	28	37	
Tumor encapsulation			
Yes	48	56	0.281
No	48	41	
Tumor differentiation			
Ⅰ- Ⅱ stage	30	23	0.241
Ⅲ – Ⅳ stage	66	74	
Liver cirrhosis			
No	11	15	0.415
Yes	85	82	
BCLC stage			
0/A	57	48	0.168
B/C	39	49	
Peritumor QSOX1			
Low	54	42	0.072
High	42	55	
Median follow-up: months	27.5	66.1	—

Abbreviations: HBsAg, hepatitis B surface antigen; AFP, α-fetoprotein; BCLC, Barcelona Clinic Liver Cancer staging system; * Fisher's exact tests.

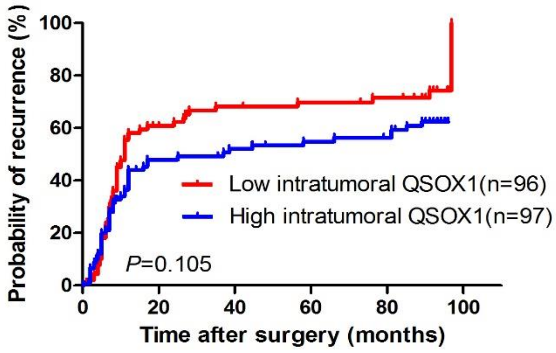

Supplementary Figure S7. Kaplan-Meier curves for time to recurrence (TTR) in HCC patients using the median intratumoral QSOX1 densities as the cutoff for the definition of subgroups.

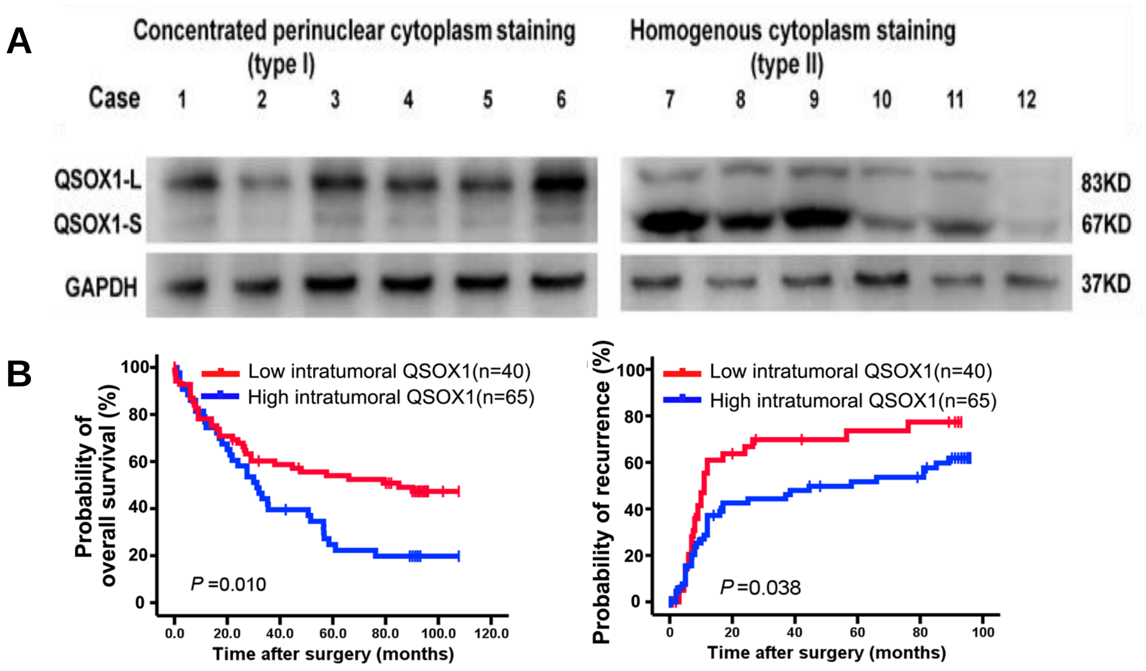

Supplemental Figure S8.The subcellular location of QSOX1 isoforms and the correlation between QSOX1-S and prognosis in HCC patients. (A) Western blot analyses of QSOX1-S and QSOX1-L expression levels in tumorous tissues with

representative type I or type II immunostaining of QSOX1 were performed. (B) Kaplan-Meier curves for overall survival and time to recurrence in a subgroup of HCC patients only with type II staining of QSOX1 in their tumor tissues according to tumorous QSOX1 level. The median QSOX1 density was used as the cutoff for the definition of subgroups.

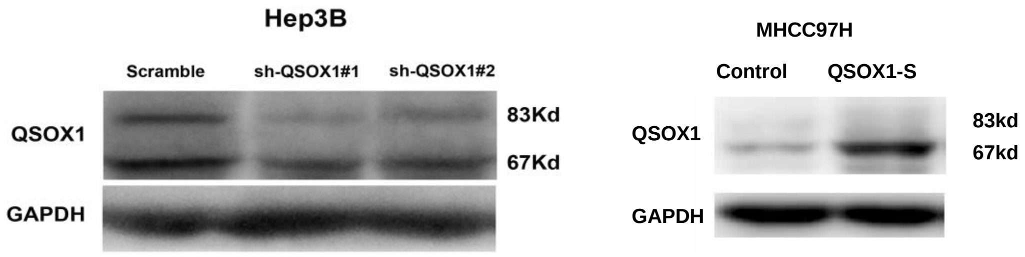

Supplementary Figure S9. QSOX1-S over-expression in MHCC97H cells and QSOX1 knockdown in Hep3B cells were validated by western bloting. (A) QSOX1 expression levels in scramble shRNA (control), sh-QSOX1#1 and sh-QSOX1#2 stably transfected Hep3B cells. Both sh-QSOX1#1 and sh-QSOX1#2 reduced QSOX1 expression levels in Hep3B cells, and Hep3B cells which was transfected by sh-QSOX1#1 were used for next functional experiments. (B) 67kd QSOX1 expression levels in QSOX1-S stably transfected MHCC97H cells and mock cells (control).

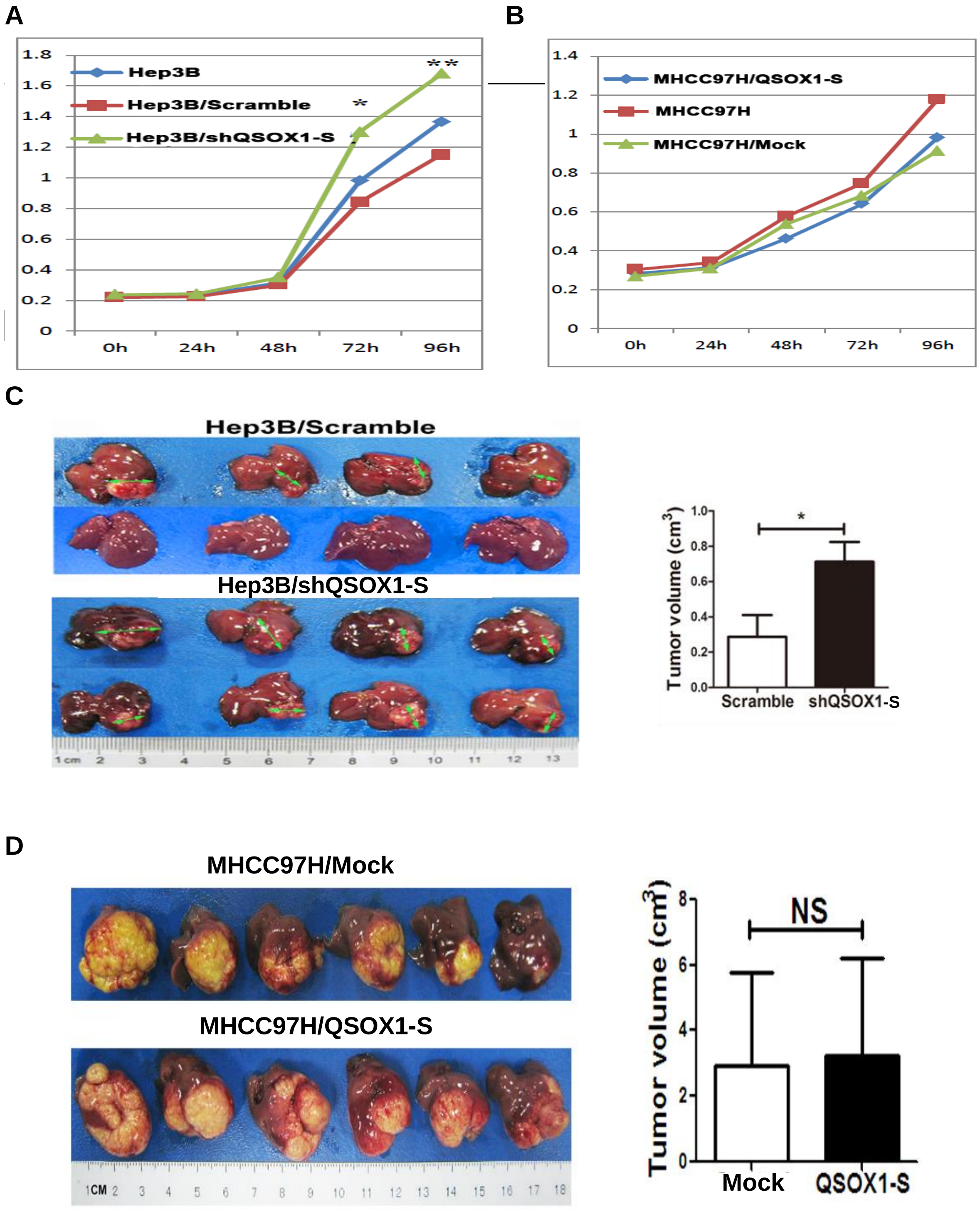

Supplementary Figure S10. The effects of QSOX1-S on *in vitro* proliferation and *in vivo* growth of HCC cells. (A) QSOX1 knockdown promoted proliferation rate of Hep3B cells (n=6). (B) The proliferation rates and cell cycle distribution of MHCC97H cells with QSOX1-S overexpression were shown (n=6). (C-D) Tumor volumes in the

orthotopic implantation models of Hep3B and MHCC97H at week 6 are shown (n =6). Values represent means ± SD.

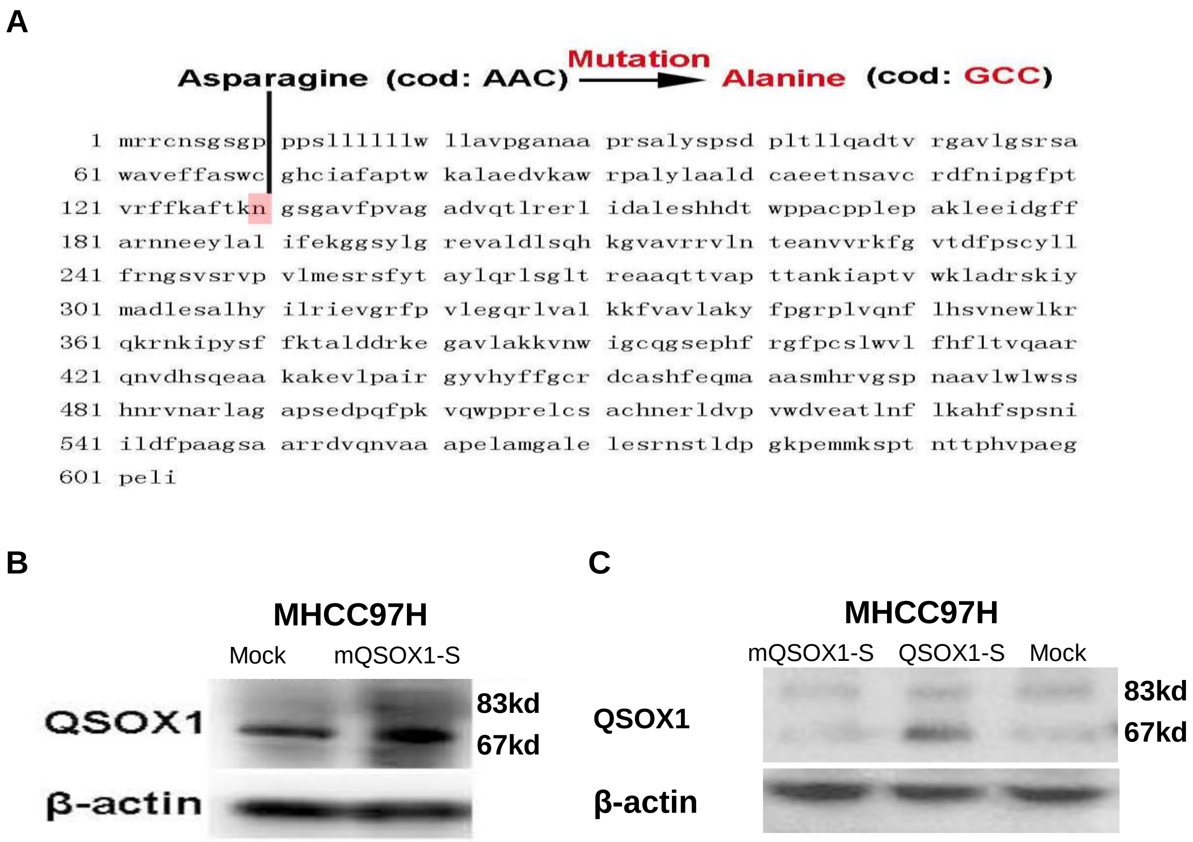

Supplementary Figure S11. The overexpression of mutant QSOX1-S in MHCC97H cell lines. (A) The plasmid for overexpressing mutant QSOX1-S (mQSOX1-S) was constructed, and Asparagine (Asn)-130 of QSOX1-S was replaced with Alanine, which results in deletion of N-glycan at Asn-130 of QSOX1-S. (B) Western blot analysis showed that 67kd QSOX1-S expression level in mQSOX1-S stably transfected MHCC97H cells was significantly increased compared to Mock cells (control). (C) To confirm the elimination of glycan at Asn-130 of QSOX1-S in

mQSOX1-S-transfected MHCC97H cells, the glycoproteins were enriched by LCA lectin affinity chromatography from mQSOX1-S- or QSOX1-S- transfected MHCC97H cells and Mock cells (control), then subjected to western blotting against QSOX1. The results from western blotting were shown. LCA-combinable QSOX1 (67kd) from mQSOX1-S-transfected MHCC97H cells was significantly decreased compared to QSOX1-S-transfected MHCC97H cells and mock cells (control), indicating core-fucosylated glycan at Asn-130 of QSOX1-S had been successfully removed in the mQSOX1-S-transfected MHCC97H cells.

Supplemental Table S5. Clinicopathological characteristics of 321 patients with HCC

Characteristic	Cohort A (n=140)			Cohort B (n=193)
	Set A (n=40)	Set B (n=40)	Set C (n=60)	
Sex: no. (%)				
Male	31 (77.5)	37 (92.5)	55(91.7)	160(82.9)
Female	9 (22.5)	3 (7.5)	5 (8.3)	33 (17.1)
Age: Mean±SD, years	53.3±12.5	51.0±10.2	50.9±9.8	52.7±10.9
HBV infection: no. (%)	40 (100)	40 (100)	60 (100)	176 (91.2)
AFP > 20 ng/ml: no. (%)	28 (70)	24 (60)	39 (65)	130(67.4)
Tumor size: Median±SD（cm）	3.3±1.3	2.5±1.2	2.5±1.7	4.5±3.3
Microvascular invasion: no. (%)	0 (0)	0 (0)	4(6.7)	65(33.7)
Tumor encapsulation: no. (%)	20 (50)	25 (62.5)	30 (50)	104 (53.9)
Tumor differentiation: no. (%)				
Ⅰ- Ⅱ stage	34(85)	27 (67.5)	39 (65)	140 (72.5)
Ⅲ - Ⅳ stage	6 (15)	13 (32.5)	21 (35)	53(27.5)
Liver cirrhosis: no. (%)	40 (100)	40 (100)	60 (100)	167 (86.5)
Child–Pugh class A: no. (%)	39(97.5)	40 (100)	59(98.3)	185(95.9)
BCLC stage: no. (%)				
0	7 (17.5)	11 (27.5)	17 (28.3)	24(12.4)
A	33 (82.5)	29 (72.5)	43 (71.7)	81 (42)
B	0 (0)	0 (0)	0 (0)	23 (11.9)
Median follow-up: months	54.5	36.8	37.3	30

*Tumor differentiation was assigned by Edmondson's grading system. Abbreviations: HBV, hepatitis B virus; AFP, alpha-fetoprotein; BCLC, the Barcelona Clinic Liver Cancer staging system.

Supplementary Table S6. Sequences for primers and shRNAs used in the study

Primers for Gene	sense sequence(5’-3’)	antisense sequence(5’-3’)
shQSOX1#1	CCGGCCGGACAATGAAGAAGCCTTTCTCGAGAAAGGCTTCTTCATTGTCCGGTTTTTG	AATTCAAAAACCGGACAATGAAGAAGCCTTTCTCGAGAAAGGCTTCTTCATTGTCCGG
sh-NC(Scramble)	CCGGAATGCCTACGTTAAGCTATACCTCGAGGTATAGCTTAACGTAGGCATTTTTTTG	AATTCAAAAAAATGCCTACGTTAAGCTATACCTCGAGGTATAGCTTAACGTAGGCATT
Wide-type QSOX1-S	AAATCTAGAATGAGGAGGTGCAACAGCG	AAAGGATCCTCAAATAAGCTCAGGTCCCTCA
Mutant QSOX1-S	CAAGGCCTTTACCAAGGGCGGCTCGGGAGCAGTAT	ATACTGCTCCCGAGCCGCCCTTGGTAAAGGCCTTG

NC, negative control

References

[1]Zhou H, Huang H, Shi J, Zhao Y, Dong Q, Jia H, et al. Prognostic value of interleukin 2 and interleukin 15 in peritumoral hepatic tissues for patients with hepatitis B-related hepatocellular carcinoma after curative resection. Gut 2010; 59:1699-708.

[2]Yang X, Liang L, Zhang XF, Jia HL, Qin Y, Zhu XC, et al. MicroRNA-26a suppresses tumor growth and metastasis of human hepatocellular carcinoma by targeting interleukin-6-Stat3 pathway. Hepatology 2013; 58:158-70.

[3]Chen P, Liu Y, Kang X, et al. Identification of N-glycan of alpha-fetoprotein by lectin affinity microarray. *J Cancer Res Clin Oncol* 2008; 134:85.
